# Postprandial hypoglycemia as a complication of bariatric and metabolic surgery: a comprehensive review of literature

**DOI:** 10.3389/fsurg.2024.1449012

**Published:** 2024-11-01

**Authors:** Mehdi Karimi, Omid Kohandel Gargari

**Affiliations:** ^1^ Faculty of Medicine, Bogomolets National Medical University (NMU), Kyiv, Ukraine; ^2^ Faculty of Medicine, Alborz University of Medical Sciences (ABZUMS), Karaj, Iran

**Keywords:** hypoglycemia, surgical complication, bariatric surgery, gastrointestinal tract surgery, review

## Abstract

Postprandial hypoglycemia (PPH) is a challenging and significant complication that can occur following bariatric and metabolic surgery. Symptoms of PPH are typical of hypoglycemia, such as sweating, weakness, disorientation, palpitation, etc. The complex nature of PPH is essential to achieve accurate diagnosis and effective management. This review aims to give extensive coverage of the intricate nature of PPH common with bariatric and metabolic surgery, outlining its pathogenesis, risk factors, clinical presentation, diagnostic strategies, and treatment options. The study explores various clinical forms and pathogenic mechanisms behind PPH while discussing diagnostic tools like continuous glucose monitoring or mixed meal tolerance tests. Furthermore, it considers possible interventions, including dietary changes, pharmaceutical therapies, and surgeries, to relieve symptoms and improve patient's quality of life. It aims to comprehensively understand how healthcare professionals can effectively manage this disorder for patients undergoing bariatric and metabolic surgery.

## 1 Introduction

Hypoglycemia after meals is known as postprandial hypoglycemia (PPH) and can induce symptoms including perspiration, weakness, disorientation, and palpitations due to low blood glucose levels (1, 2). It is a serious complication, particularly in patients who have undergone upper gastrointestinal (GI) tract surgery, such as bariatric and metabolic surgery, because it can have a significant impact on GI physiological functions, recovery, quality of life, and patients’ health. This disorder involves elevated insulin production during meals, which causes modest to severe hypoglycemia. Appropriate management and treatment options are required to improve patient outcomes and quality of life (3–6).

In recent years, there has been growing recognition of the prevalence and significant impact of PPH in patients who have undergone bariatric and metabolic surgery (4). Despite its clinical importance, PPH must still be better understood and under-researched. With the increasing incidence of upper GI disorders, the challenges in diagnosis, and the negative impact on patient well-being, PPH represents a significant concern in gastroenterology. This led to increased interest in understanding its underlying mechanisms, identifying those most at risk, and determining practical management approaches. However, the available information could be more cohesive and consistent, making it easier for healthcare providers to synthesize and apply it effectively.

This review seeks to provide a thorough summary of PPH following bariatric and metabolic surgeries, covering its frequency, symptoms, diagnostic techniques, and treatment choices. By consolidating this information, the objective is to enhance comprehension, improve clinical decision-making, and ultimately achieve better outcomes for patients grappling with this complex condition.

## 2 Review of bariatric and metabolic surgeries

Bariatric surgery remains the most effective long-term treatment for morbid obesity type-2 diabetes and metabolic syndrome, with recent updates expanding eligibility and improving safety and outcomes (7). In this section, we review the four main types of these surgeries.

### 2.1 Sleeve gastrectomy (SG)

Sleeve gastrectomy (SG) is one of the most commonly performed bariatric surgical procedures aimed at inducing body mass loss and improving metabolic profile in patients with morbid obesity. In this procedure, 75%–80% of the stomach is laparoscopically removed, forming a sleeve-like organ that limits food consumption and satiety-inducing changes in gut hormones (8). SG is highly effective for achieving clinically significant weight loss, with an average total body weight loss of 23% in one year and 16% following five years postoperatively (9, 10). While SG is technically less complex than RYGB and safer in general, it may be inferior to RYGB for treating some comorbid conditions, including gastroesophageal reflux disease (GERD). Despite its benefits, SG is associated with hazards such as leaks, nutritional inadequacies, and the development of GERD, emphasizing the significance of long-term follow-up and lifestyle changes for long-term success (11–10).

### 2.2 Roux-en-Y gastric bypass (RYGB)

Roux-en-Y Gastric Bypass (RYGB) is an established bariatric procedure that delivers weight loss and improves metabolic health, particularly for patients with obesity and its comorbidities, such as type 2 diabetes (12). This procedure involves creating a small stomach pouch and attaching it directly to the lower part of the small intestine, bypassing most of your stomach and upper duodenum and reducing calorie absorption (13). RYGB is an effective operation that results in substantial weight loss and superior glycemic control compared to intensive lifestyle management. It has broader metabolic effects that reduce the risk of long-term renal impairment and cardiovascular risk factors. While RYGB may be superior to other bariatric procedures, including sleeve gastrectomy for glycemic control and triglyceride reduction, the SG might lead to a more significant BMI (14, 15). RYGB is associated with long-term weight loss and co-morbidity resolution but carries the risks of malnutrition and gastrointestinal adverse events, necessitating judicious patient selection in conjunction with life-long follow-up for optimal outcomes (16).

### 2.3 One anastomosis gastric bypass (OAGB)

One Anastomosis Gastric Bypass (OAGB) is a bariatric surgery that promotes weight loss and improves metabolic health, serving as a simpler alternative to the traditional RYGB (17, 18). This procedure involves creating a small gastric pouch and connecting it directly to the small intestine, bypassing a portion of the stomach and initial small intestine. OAGB is effective in achieving significant weight loss and addressing obesity-related conditions like type 2 diabetes and hypertension. It is generally safer with fewer complications than other bariatric surgeries, though risks include nutritional deficiencies, bile reflux, and marginal ulcers (19, 20). Additionally, OAGB can be used as a revisional surgery for patients with inadequate results from previous procedures. Compared to RYGB, OAGB offers similar outcomes with a simpler surgical technique, making it a viable option for those seeking bariatric surgery, provided there is careful patient selection and diligent postoperative management (20).

### 2.4 Single anastomosis duodeno-ileostomy with sleeve gastrectomy (SADI-S)

Single Anastomosis Duodeno-ileostomy with Sleeve Gastrectomy (SADI-S) is a bariatric surgery that effectively combines restrictive and malabsorptive techniques to promote significant weight loss and metabolic improvement. By creating a single connection between the duodenum and ileum, this procedure bypasses a large portion of the small intestine, reducing nutrient absorption (21, 22). SADI-S is especially beneficial as a revisional surgery for patients who have experienced insufficient weight loss or regain after an initial sleeve gastrectomy, showing favorable results compared to other bariatric procedures. While effective, SADI-S requires careful postoperative management due to potential complications such as nutritional deficiencies and gastrointestinal issues (23).

### 2.5 Comparison

Table 1 summarizes and compares the four main types of bariatric and metabolic surgeries. The choice of procedure depends on individual patient needs, comorbidities, and surgical goals. Long-term follow-up and lifestyle changes are crucial for the success of any bariatric surgery.

**Table 1:** Comparison of four main types of bariatric and metabolic surgery.

	SG	RYGB	OAGB	SADI-S
Procedure	Removes a large portion of the stomach, creating a sleeve shape	Creates a small stomach pouch and bypasses part of the small intestine	Creates a small gastric pouch with a single intestinal connection	Involves SG and a single connection to the ileum
Efficacy	Effective for weight loss and metabolic improvements	Highly effective for weight loss and diabetes improvement	Effective with simpler surgical technique than RYGB	Effective, especially as a revisional procedure
Metabolic Benefits	Improves metabolic parameters, but less effective for GERD	Significant improvements in diabetes and metabolic syndrome	Similar metabolic benefits to RYGB	Offers metabolic benefits, particularly in revisional cases
Safety and Complications	Risk of leaks, nutritional deficiencies, and GERD	Risk of nutritional deficiencies and gastrointestinal issues	Lower complication rates, but risks include bile reflux	Potential for nutritional deficiencies and diarrhea
Long-term Outcomes	Sustained weight loss, but GERD may persist	Sustained weight loss and metabolic improvements	Sustained weight loss with fewer complications	Effective long-term weight loss, especially after previous surgeries
Revisional Use	Often a primary procedure, but can precede other surgeries	Can be used for revisional surgery	Used as a revisional option for previous surgeries	Commonly used as a revisional procedure after SG

OAGB, one anastomosis gastric bypass; RYGB, Roux-en-Y gastric bypass; SADI-S, single anastomosis duodeno-ileostomy; SG, sleeve gastrectomy.

## 3 Incidence and prevalence of PPH

Different studies have reported widely varying incidence rates for PPH, ranging from 10% to 72%. The wide range is due to the differences in study populations, diagnostic criteria, follow-up durations, and timing of assessment, as well as evaluation tools used (24–26). These figures are based on patients’ admission notes or self-reports concerning related symptoms (27). Except for some case studies, there are no published incidence rates of hypoglycemia following bariatric and metabolic surgery (4, 28). Studies have reported that the incidence of PPH varies depending on the type of surgery undergone (25, 29). Over five years, a study found that the occurrence of hyper-insulinemic PHH after RYGB surgery started at 0.5% before the surgery. It then increased to 9.1% at 12 months post-surgery and slightly decreased to 7.9% at 60 months (5 years) after the surgery (30).

A randomized trial comparing SG to RYGB found that 14% of SG patients had reactive hypoglycemia (blood glucose <3.1 mmol/L after 75-g oral glucose load) one year after surgery. This implies that, while hypoglycemia can occur after SG, it may be less common than after RYGB (29). A retrospective clinical study found that the incidence of dumping syndrome after OAGB (42.9%) was lower than that observed after RYGB (56.4%) but significantly higher than after SG (15.6%) (29). A study indicated that over time, revealing that the cumulative occurrence of RYGB hypoglycemia rose from 2.7% to 13.3% between the first- and fifth years post-surgery. The PPH following bariatric and metabolic surgery might probably be associated with a lower preoperative body mass index (BMI), reduced levels of HbA1c, and a higher percentage of excess weight loss (31).

## 4 Pathophysiological mechanisms of PPH

Previous studies have suggested some theories that the basic pathophysiological mechanisms of PPH after upper bariatric and metabolic surgeries may include hypersecretion of incretin, sensitivity or resistance to insulin, dysregulation of the “intrapancreatic axis,” and alpha-cell dysfunction. However, the exact pathophysiological mechanism of PPH is unclear (25, 32–34).

The exact mechanisms of PPH following bariatric and metabolic surgeries are not fully understood, but several hypotheses have been proposed:

1) *Incretin hypersecretion*: One of the leading hypotheses is the exaggerated secretion of incretin hormones, primarily glucagon-like peptide-1 (GLP-1) and glucose-dependent insulinotropic polypeptide (GIP), in response to the accelerated delivery of nutrients to the small intestine after these surgeries (25, 35, 36). GLP-1 secretion significantly increases after RYGB and, to a lesser extent, after SG compared to non-operated individuals (36). GIP secretion is lowest after RYGB but remains elevated after SG (36). The exaggerated incretin response, especially GLP-1, leads to an inappropriate and excessive stimulation of insulin secretion, resulting in postprandial hyperinsulinemic hypoglycemia (25, 35).
2) *Changes in insulin kinetics*, including increased insulin secretion, decreased hepatic insulin clearance, and altered insulin sensitivity, have been implicated in PPH (37, 38). Increased beta-cell glucose sensitivity and insulin secretion have been observed after both RYGB and SG (36, 39). Diminished hepatic insulin extraction may contribute to higher circulating insulin levels and hypoglycemia risk (37). Rapid weight loss and changes in insulin sensitivity may also play a role in the development of PPH (25).
3) *Dysregulation of Other Hormones*. Disturbances in regulating other hormones, such as glucagon, glicentin, and ghrelin, have been proposed as potential contributors to PPH. Alpha-cell dysfunction and impaired glucagon secretion may exacerbate PPH. Increased postprandial secretion of glicentin, a marker of PPH risk, has been observed in some studies. Disrupted negative feedback between insulin and ghrelin may contribute to the pathogenesis of PPH (25, 35, 40).
4) *Anatomical and Physiological Changes.* The altered anatomy and physiological changes following bariatric and metabolic surgeries, such as accelerated gastric emptying, intestinal transit time, and nutrient absorption, are thought to play a role in developing PPH. Rapid gastric emptying and nutrient delivery to the small intestine after RYGB may contribute to the exaggerated incretin and insulin responses. Changes in gut hormone secretion patterns and intestinal adaptation after surgery may also be involved (41, 42).

The rapid influx of carbohydrates into the small intestine following bariatric surgery increases the release of incretin hormones such as GLP-1. This excessive secretion of insulin by the pancreas in reaction to the incretins is a significant contributor to postprandial hyperinsulinemic hypoglycemia (43). Increased tissue sensitivity to insulin, mediated by factors like insulin-like growth factor-1, can also promote the development of hypoglycemia (25). Besides the incretin effect, nutrient passage through the GI tract may trigger harmful feedback mechanisms (anti-incretins) to counterbalance the effects of glucose-lowering. Alterations in this balance, resulting from bypassing the duodenum, jejunum, and a portion of the ileum during bariatric procedures, can induce postprandial hyperinsulinemic hypoglycemia (33).

Postoperative metabolic hypoglycemia is partly caused by altered gastric emptying of ingested food, resulting in rapid glucose absorption in the intestine and extreme postprandial secretion of GI peptides, particularly GLP-1 (4). Mismatches between the time of insulin secretion and glucose absorption (44) or insulin over-secretion (45) are the main known reasons, which seem to be multifactorial, but the primary regulator is interleukin 1-β (46). The rise of incretin hormones such as GIP (44) and GLP-1 was reported in many studies after gastric bariatric surgery (34, 44, 47–49) and vagotomy subjects with pyloroplasty (50).

Rapid weight loss and regression of insulin resistance after bariatric surgery may lead to a slower normalization of insulin production, contributing to hypoglycemia. Changes in the activity of pancreatic alpha cells, responsible for glucagon secretion, may also contribute to the onset of PPH (25).

GLP-1 is hypersecretion from L cells (50) and hypertrophies β cells via enhanced expression of the transcription factor of islet cells and duodenal homeobox-1 protein (47). The process of hypertrophy and hyperplasia of β cells has been named nesidioblastosis (51, 52) which has been known to have a role in hyperinsulinemia (51–53). However, this finding has not been seen in most reported cases (49, 54).

**Figure 1:**
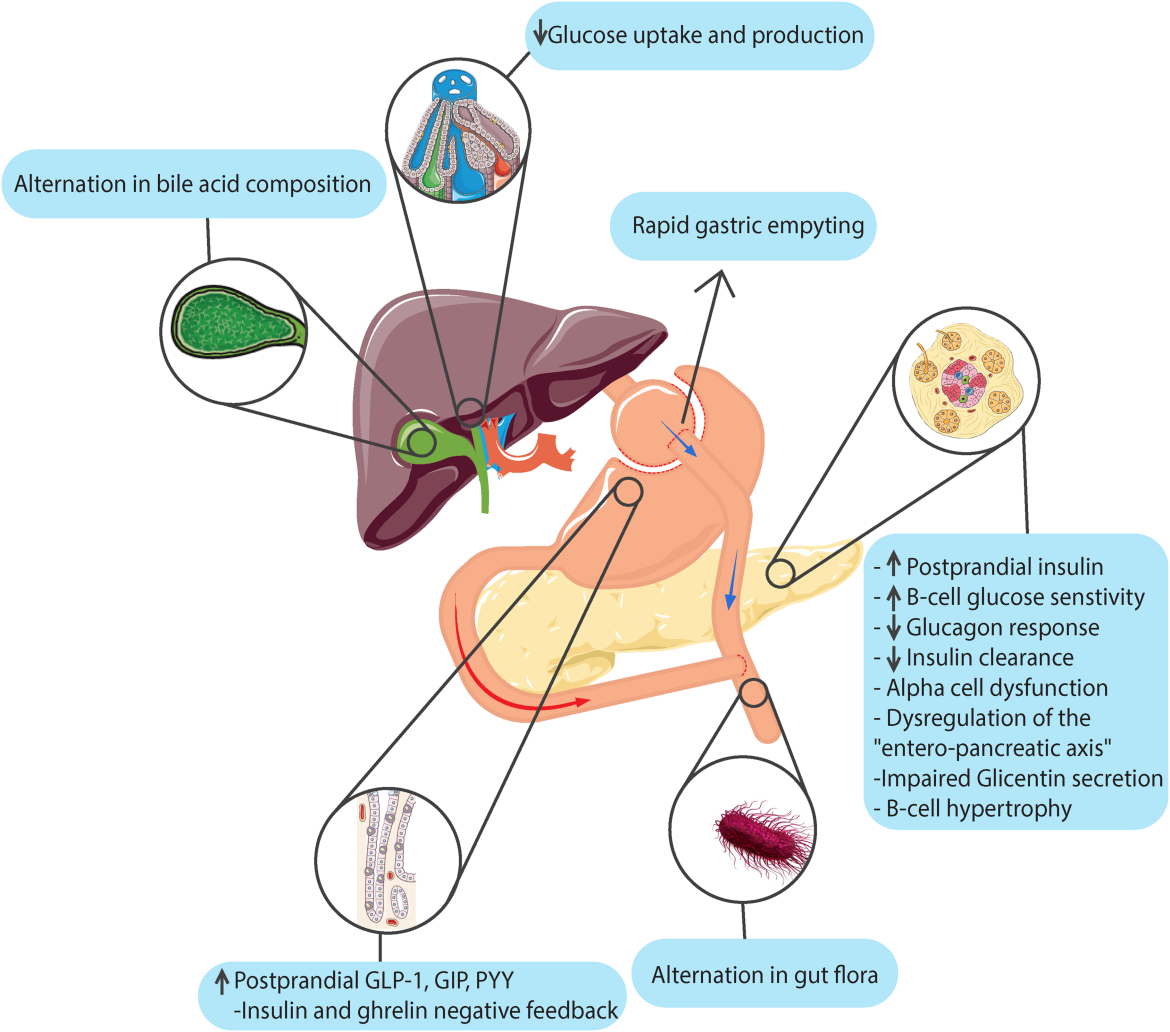
Etiological mechanisms of hypoglycemia following bariatric and metabolic surgeries. (This image is designed and generated by the authors).

Figure 1 illustrates a summary of the etiological mechanisms of hypoglycemia that can occur following bariatric and metabolic surgeries.

## 5 Risk factors for PPH following bariatric and metabolic surgery

Preoperative factors include lower BMI, higher insulin sensitivity, preoperative hypoglycemic symptoms, female sex (34, 55, 56). Studies have found that patients with a lower preoperative BMI are at a higher risk of developing PPH after RYGB and SG. This association may be related to higher insulin sensitivity in leaner individuals (55, 56). Patients with higher preoperative insulin sensitivity, measured by indices like the oral glucose insulin sensitivity index, are more likely to experience PPH after RYGB and SG (55). The presence of preoperative symptoms suggestive of hypoglycemia has been identified as a significant risk factor for developing PPH after bariatric surgery (31). Several studies have reported a higher prevalence of PPH symptoms in female patients after RYGB and SG (34, 56).

Surgical factors include the bariatric procedure type and the time since surgery. RYGB has been consistently associated with a higher risk of PPH compared to SG. The incidence of severe hypoglycemic episodes requiring hospitalization is also higher after RYGB. A longer duration since the bariatric surgery has been linked to an increase (31, 34, 57)

Overall, risk factors associated with PPH following bariatric and metabolic surgeries as identified in retrospective epidemiological studies comprise female gender, younger age, absence of diabetes diagnosis before surgery, history of pre-surgery hypoglycemia unrelated to diabetes or diabetes medications, lower pre-surgery hemoglobin A1C (HbA1C) levels, and increased excess weight loss after the operation (31, 58). Patients with reduced BMI after bariatric surgery are at increased risk of postprandial hyperinsulinemic hypoglycemia, particularly those with high insulin secretion and beta-cell function pre-surgery. Younger individuals and those undergoing upper bariatric and metabolic surgery are more susceptible. Rapid weight loss, improved insulin sensitivity post-surgery, and faster carbohydrate absorption and incretin hormone imbalance contribute to this risk (38, 59, 60).

It has been shown that elevated pre-surgery plasma glucose levels, increased insulin sensitivity, and heightened beta-cell glucose sensitivity are significant predictors of spontaneous self-reported PPH following RYGB and laparoscopic SG (55). Younger age, lower preoperative BMI, and high postprandial beta-cell activity are associated with a higher risk of developing PPH (58).

Table 2 presents the risk factors for PPH following bariatric and metabolic surgeries.

**Table 2:** The risk factors for PPH following bariatric and metabolic surgeries.

Risk factors	Participants	Ref.
Preoperatively: - Lower weight Postoperatively: - Higher weight loss - Higher insulin sensitivity - Increased β-cell function	Healthy patients four years after RYGB	(61)
Preoperatively: - Lower BMI - Lower HbA1C Postoperatively: - Excess weight loss during 6 months	Non-diabetic patients PPH following RYGB1	(31)
Preoperatively: - Lower BMI - Lower fasting glucose - Higher insulin sensitivity - Higher β-cell glucose sensitivity Postoperatively: - Higher glucose peak on the OGTT	Obese non-diabetic patients PPH following RYGB or laparoscopic SG	(55)
- Female gender - Present hypoglycemic symptoms preoperatively - RYGB surgery - Years since surgery	Healthy patients following RYGB or vertical SG	(57)

PPH, postprandial hypoglycemia; BMI, body mass index; RYGB, Roux-en-Y gastric bypass; SG, sleeve gastrectomy; OGTT, oral glucose tolerance test (31).

## 6 Clinical manifestations and complications of PPH following bariatric and metabolic surgery

Clinical manifestations in PPH following bariatric and metabolic surgeries depend on the severity of hypoglycemia. Heart palpitations, anxiety and disorientation, hunger, perspiration, excitation, tremors, and paresthesia are among the unsettling symptoms that mild to moderate hypoglycemia can produce. In contrast, severe hypoglycemia may manifest as drowsiness, delirium, disorientation, seizures, and comes (62, 63). Early dumping symptoms, which might include diarrhea, palpitations, lightheadedness, extreme weariness, nausea, and vomiting, usually appear 10–30 min after a meal. Usually, glucose levels are not low at the onset of these symptoms (48). These symptoms can be seen when the blood glucose is less than 55 mg/dl. However, this scale can be shifted lower when a person currently has hypoglycemia (52).

Hypoglycemia symptoms are nonspecific, making differential diagnosis crucial. These symptoms fall into two categories: neuroglycopenic and autonomic. *Neuroglycopenic* symptoms arise from central nervous system deprivation and range from mild (e.g., blurred vision, dizziness, flushing, drowsiness, fatigue, weakness) to severe (e.g., seizures, loss of consciousness, confusion, difficulty speaking). *Autonomic* symptoms result from the activation of the autonomic nervous system and are divided into *adrenergic* (e.g., shakiness, heart-pounding, anxiety) and cholinergic (e.g., sweating, hunger, paresthesia) (64) (Table 3).

**Table 3:** Signs and symptoms of hypoglycemia.

Autonomic symptoms	Neuroglycopenic symptoms
i. Adrenergic symptoms - Shakiness or Tremors - Palpitations - Anxiety - Profuse Sweating - Pallor (Pale skin) - Cold, Clammy Skin ii. Cholinergic symptoms - Sweating - Hunger - Paresthesia - Nausea	• Cognitive impairment: - Confusion - Difficulty concentrating - Memory lapses - Slurred speech • Behavioral changes: - Irritability - Mood swings - Unusual behavior or personality changes • Motor symptoms: - Weakness - Lack of coordination - Difficulty walking • Visual disturbances: - Blurred vision - Double vision • Severe symptoms: - Seizures - Loss of consciousness - Coma

Severe PPH can lead to severe neuroglycopenic symptoms such as seizures, disorientation, loss of consciousness, and even hypoglycemic coma. These neuroglycopenia symptoms often occur with fainting spells, especially after large meals, and can be mistaken for other conditions, necessitating careful diagnostic workup (65). Severe hypoglycemia also may cause motor vehicle accidents, falls, and even death. Associated disability and loss of quality of life and this situation cannot be healed during this time (66).

### 6.1 Dumping syndrome

Dumping syndrome is a disorder that may develop following bariatric and metabolic surgery, which occurs when food, particularly sugar, passes from the stomach to the small colon too rapidly. There are two forms of dumping syndrome: early and late dumping syndrome (67–69).

Early dumping syndrome occurs 10 to 30 min after eating. It presents with symptoms like nausea, vomiting, abdominal cramps, diarrhea, dizziness, and rapid heart rate, resulting from the rapid movement of food into the small intestine, causing fluid shifts and blood pressure changes. It is commonly seen after surgeries like gastrectomy or gastric bypass, which disrupt normal stomach function and emptying. Management typically involves dietary adjustments, such as eating smaller, more frequent meals, avoiding high-sugar foods, and increasing fiber intake, with medications used in some cases to slow gastric emptying (67, 69, 70).

Late dumping syndrome, which occurs 1 to 3 h after eating, is characterized by hypoglycemia, weakness, sweating, dizziness, and disorientation caused by an increased insulin response owing to fast sugar absorption in the small intestine (71–73). It is associated with procedures that alter stomach function, similar to early dumping syndrome, but the essential distinction is in time and the insulin-related mechanism (72). Management includes dietary changes such as preferring complex carbs, increasing protein intake, and avoiding fluids with meals. In some situations, medications such as acarbose or diazoxide may be needed. Both early and late dumping syndromes may have a major impact on quality of life, but with adequate therapy, symptoms are typically efficiently managed (74).

## 7 Diagnosis of PPH following bariatric and metabolic surgeries

### 7.1 Clinical considerations

Currently, there are no established clinical guidelines for diagnosing PPH. Collecting a detailed disease history is crucial, and provocative tests have been proposed for detection. A comprehensive clinical history and physical examination can help identify the underlying reason and guide further diagnostic tests. Non-diabetic hypoglycemia should be evaluated and managed individually depending on clinical symptoms and probable diagnosis (75). Patients show postoperative episodes of hypoglycemia with adrenergic, cholinergic, and neuroglycopenic signs and symptoms (45, 76). *Whipple's triad* is a diagnostic tool with specific parameters to identify hypoglycemia. The three components of hypoglycemia are symptoms, hypoglycemia, and relief after rising plasma glucose concentration (77).

Besides venous blood glucose testing, several other diagnostic techniques can be useful to diagnose PPH, such as continuous glucose monitoring (CGM), Glycemic pattern, Histopathology, Selective arterial calcium stimulation test, and Radiological investigation (78).

Based on specific blood glucose level thresholds, the seriousness of hypoglycemia is classified as (79, 80):

i. Mild hypoglycemia: Blood glucose levels between 54 and 70 mg/dl (3.0–3.9 mmol/L)
ii. Moderate hypoglycemia: Blood glucose levels between 40 and 54 mg/dl (2.2–3.0 mmol/L)
iii. Severe hypoglycemia: Blood glucose levels ≤40 mg/dl (2.2 mmol/L)

### 7.2 Blood glucose testing

Blood glucose testing is essential for diagnosing and managing this condition. Standard tests in actual practice include the oral glucose tolerance test (OGTT), the mixed meal tolerance test (MMTT) (63), and continuous glucose monitoring (CGM) (81, 82).

The OGTT is commonly used to diagnose hypoglycemia. It involves administering a 75-g glucose load and measuring blood glucose levels at various intervals. However, this test can sometimes lead to over-diagnosing improved glucose tolerance due to postprandial hyper-insulinemic hypoglycemia observed in many post-surgery patients (83).

Another method is the Mixed Meal Tolerance Test (MMTT), which uses a meal containing carbohydrates and fats equivalent to 75 g of glucose. It is considered more reflective of real-life conditions than the OGTT. The MMTT has shown that post-surgery patients often do not exhibit hypoglycemia, indicating any improvement in glucose tolerance compared to pre-surgery data (83, 84).

Continuous glucose monitoring (CGM) is increasingly used to diagnose and manage PPH. It provides continuous data on glucose levels, capturing fluctuations that might be missed with intermittent point-of-care (POC) blood glucose checks. CGM has been essential in detecting asymptomatic hypoglycemia and glycemic excursions in pediatric and adult patients’ post-surgery (81, 82, 84).

Studies showed that the MMTT effectively detects PPH and severe hypoglycemic events, particularly in patients with persistent post-bariatric hypoglycemia during long-term follow-up. CGM complements the MMTT by identifying asymptomatic hypoglycemia, fasting hypoglycemia, and glucose variability over an extended period. Combining both tests may provide the most comprehensive assessment for diagnosing persistent post-bariatric hypoglycemia (84, 85). Maia et al. showed that the CGMS effectively detects PPH and improves therapeutic management but has low sensitivity to detect unrecognized hypoglycemia in type 1 diabetes patients (86). Baseline parameters, such as HbA1c and weight loss, can help predict PPH in patients after gastric bypass surgery, aiding in screening and selecting those requiring further evaluation (46).

Venous samples are recommended for testing glucose concentration because capillary blood glucose can falsely be lower in the setting of relative hypotension and Raynaud’s disease (78).

### 7.3 Diagnostic medical imaging

Diagnostic medical imaging plays a crucial role in evaluating and differentially diagnosing PPH after upper GI surgery, particularly in ruling out other causes of hypoglycemia, like insulinoma, as the underlying cause (87).

The diameter of the gastroenterostomy has a considerable impact on quick stomach emptying, which is a crucial determinant in the development of PPH (88). This connection must be considered while doing medical imaging since a bigger diameter may result in faster food transit into the jejunum. This expedited process might cause an excessive insulin response, which contributes to the symptoms of PPH (89). Understanding the gastroenterostomy's features during imaging examinations can help effectively identify and treat individuals with postprandial hypoglycemia.

CT volumetry is a diagnostic imaging technique used to evaluate anatomical changes after bariatric and metabolic surgery (90). It primarily assesses gastric reservoir capacity and its association with clinical outcomes such as weight loss and problems. However, according to current literature, its direct relevance in identifying PPH after bariatric surgery is not well-established (87, 90).

Computed Tomography (CT) Scan is often the initial imaging modality to assess the pancreas and surrounding structures for potential insulinomas or other pancreatic lesions. However, CT scans may not detect small insulinomas, limiting their diagnostic utility (91, 92).

Endoscopic ultrasound (EUS) is the most sensitive imaging technique for detecting small pancreatic lesions, including insulinomas. It allows for high-resolution visualization of the pancreas and can guide fine-needle aspiration (FNA) for cytological evaluation if a suspicious lesion is identified (92).

Selective arterial calcium stimulation test (SACST) is an invasive procedure that involves injecting calcium gluconate into the arteries, supplying the pancreas with insulin to stimulate insulin release from potential insulinomas. It can help localize the source of excessive insulin production when imaging is inconclusive (93).

## 8 Management and treatment of PPH after upper GI surgery

**Figure 2:**
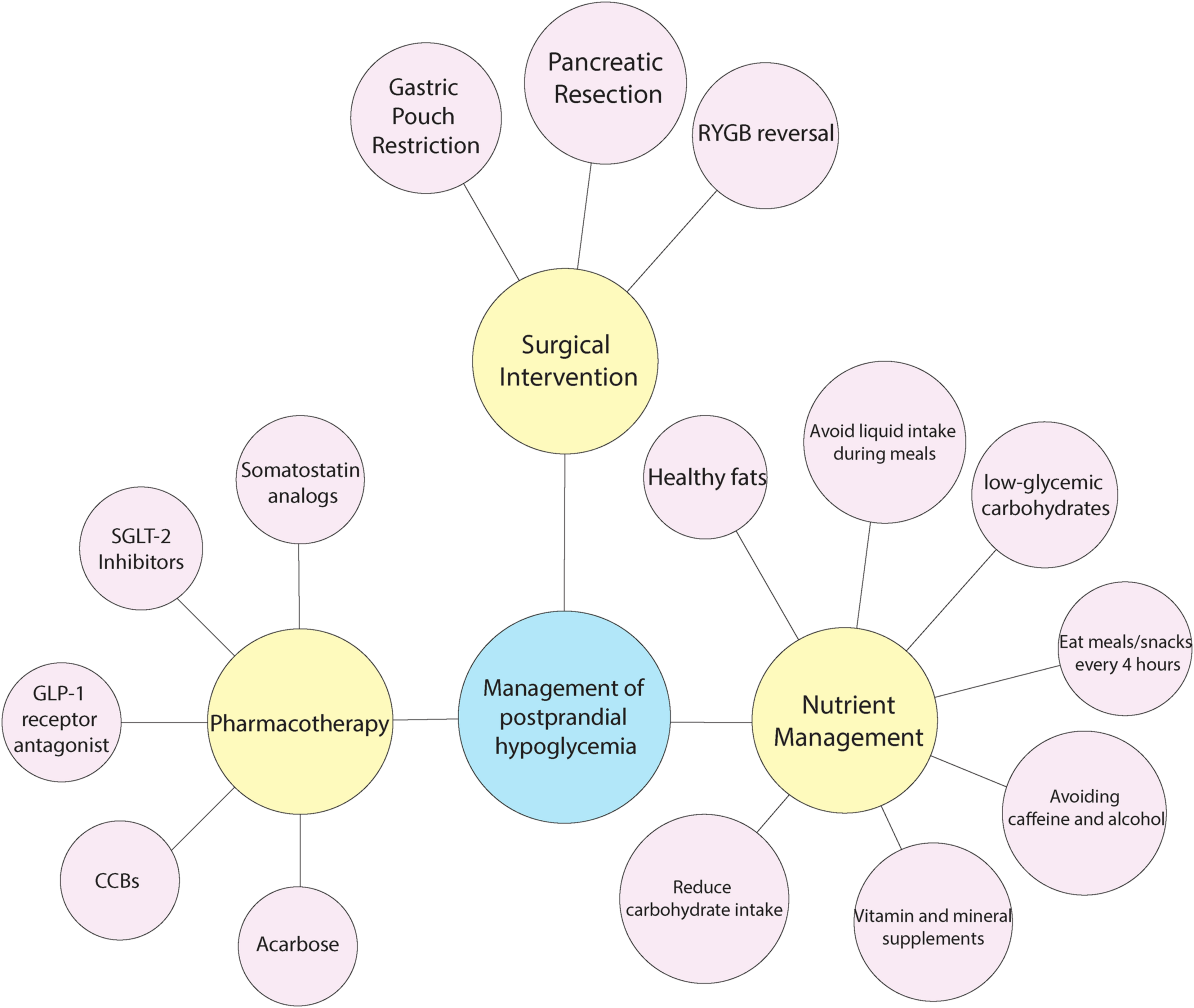
Management of PPH.

Managing PPH after upper GI surgery is challenging, aimed at stabilizing blood sugar levels and preventing sharp drops that lead to hypoglycemia. Practical strategies for managing PPH, especially in individuals who have undergone bariatric surgery, include a combination of lifestyle changes, exercise, medication, and surgical interventions. A personalized, multidisciplinary strategy tailored to each patient's specific requirements is crucial for successfully managing PPH (53, 94). (see Figure 2).

### 8.1 Lifestyle modifications and exercise

Postprandial exercise has been studied for its effects on glucose levels. A study by Ternhamar et al. concluded that moderate-intensity exercise shortly after meal intake did not significantly lower plasma nadir glucose levels in RYGB patients. However, replacing high-glycemic-index meals with low-glycemic-index meals showed some benefit in reducing glucose excursions (95).

### 8.2 Nutrition management

*Nutritional management* is defined as “Adjusting the quantity and quality of food intake to improve an individual's health status (96). Accordingly, nutritional therapy for post-bariatric hypoglycemic patients aims to reduce rapid glucose rise after meal consumption (97). Patients are often advised to consume small, frequent meals with low glycemic index carbohydrates combined with proteins and fats to manage PPH (32). It is known that high-carbohydrate, low-protein meals cause hypoglycemia more strongly (98). Therefore, hypoglycemia is associated with the type of food consumed, and dietary modifications could be a possible treatment for post-prandial hypoglycemia. Eating small but numerous meals is an appropriate option also for pregnant patients (99). It is proved that reducing the amount of carbohydrates, combined with a higher protein consumption, lowers the risk of hypoglycemia by decreasing insulin secretion (100). Generally, most hypoglycemic patients (mild to moderate cases) are supposed to be cured with regimen adjustments (55, 101, 102). However, severe cases do not respond to diet modifications (103).

Suhl et al. (97) studied medical nutrition therapy in post-prandial hypoglycemic patients. They indicate 10 points for nutritional management, including consumption of low-glycemic carbohydrates, healthy fats, high but calculated amounts of protein, and avoiding caffeine and alcohol. They emphasize vitamin and mineral supplements. In addition, patients should avoid liquid intake during meals and eat meals/snacks every 4 h. A case series by Abrahamsson et al. (47) approves this point. They also suggest a low-carbohydrate, protein-rich diet before starting pharmacotherapy. Other studies focus mainly on the amount of carbohydrates. It is known that limiting carbohydrate intake and eating multiple meals is a successful dietary modification (104).

Applying nutritional management for hypoglycemic patients has some difficulties; for example, hyperinsulinemic hypoglycemic patients’ tendency towards carbohydrate consumption increases (105). Furthermore, most patients need better nutritional knowledge and undesirable food habits. These factors affect the success of nutritional management and should be considered for the patients’ management (94, 97, 106).

### 8.3 Pharmacological therapy

Pharmacotherapy is essential for managing PPH following bariatric and metabolic surgery when lifestyle modifications and dietary changes are insufficient. It modulates insulin secretion, delays carbohydrate absorption, stabilizes blood glucose levels, and alleviates symptoms (107). Pharmacotherapy offers several options for managing PPH. The choice of medication depends on the specific pathophysiological mechanism and the patient's overall health. Combining medication with dietary changes and continuous glucose monitoring can effectively manage this condition. Close monitoring by a healthcare professional is essential to adjust treatment plans and manage any potential side effects.

Numerous studies showed that these pharmacological groups have potential therapeutic effects on PPH. They are SGLT2 Inhibitors and IL-1 Antagonists (108–110)., GLP-1 Receptor Antagonists (47, 111, 112), GLP-1 Receptor Agonists (47, 111) Calcium Channel Blockers, and Acarbose (113), Somatostatin Analogs (114), and Diazoxide (115).

- *SGLT2 Inhibitors and IL-1 Antagonists: Empagliflozin*, an *SGLT2 inhibitor*, and *Anakinra*, an IL-1 receptor antagonist, both significantly reduce postprandial insulin release and prevent hypoglycemia in patients after gastric bypass surgery (108–110).
- *GLP-1 Receptor Antagonists:* GLP-1 receptor antagonists, such as exendin (11–39), correct hypoglycemia by reducing postprandial insulin secretion and stabilizing glucose levels in patients with hyperinsulinemic hypoglycemia after gastric bypass (47, 111, 112).
- *GLP-1 Receptor Agonists:* GLP-1 receptor agonists have shown potential in managing PPH by stabilizing glucose levels without causing hypoglycemia, although more controlled studies are needed to confirm their efficacy (47, 111).
- *Calcium Channel Blockers and Acarbose:* Verapamil, a calcium channel blocker, and acarbose, an alpha-glucosidase inhibitor, have been used to reduce the frequency and severity of hypoglycemic episodes in patients with non-insulinoma pancreatogenous hypoglycemic syndrome (NIPHS) after bariatric surgery (113)
- *Somatostatin Analogs:* Octreotide, a somatostatin analog, has effectively managed postprandial hyperinsulinemic hypoglycemia by attenuating the exaggerated postprandial insulin and incretin response, leading to significant symptom relief (114).
- *Diazoxide:* Diazoxide, a KATP channel opener, has successfully managed severe PPH in patients after RYGBby reducing insulin secretion (115).

Hepprich et al. showed that SGLT2-inhibitors and IL-1 antagonism may improve PPH after gastric bypass surgery by reducing glucose-induced IL-1 and preventing hypoglycemia (108).

The PID algorithm accurately and safely adjusts glucose infusion rate for post-prandial hypoglycemic clamps in both healthy and bariatric surgery patients, ensuring standardized results (116).

### 8.4 Surgical interventions

Surgical interventions for PPH, such as gastric bypass reversal, partial gastrectomy, or pancreatic resection, are typically considered only when non-surgical treatments like dietary modifications, pharmacotherapy, and continuous glucose monitoring have failed. These surgeries aim to address severe cases by altering the GI anatomy or managing excessive insulin production, but they carry significant risks and are usually reserved for the most refractory cases (53, 117). Surgical procedures will slow the gastric reserve's rapid transit to the intestine or restore the GI system to its typical structure (118).

Surgical intervention for severe post-RYGB hypoglycemia includes pancreatic resection, RYGB reversal, and gastric pouch restriction, with resolution of symptoms in 67%, 76%, and 82% of patients, respectively (119, 120).

Gastric pouch restriction is the most commonly performed surgical treatment for PPH after RYGB. Treatment options include procedures like pouch banding and/or pouch resection, which aim to control the size of the gastric pouch and reduce the severity of hypoglycemic episodes (121).

In rare circumstances, a partial gastrectomy may be done to lower the size of the stomach pouch, which can assist delay gastric emptying and minimize the risk of hypoglycemic episodes. However, this technique is more intrusive and has serious dangers (53).

Partial pancreatic resection, though controversial and typically reserved for severe cases, has been considered in managing PPH linked to hyperinsulinemia caused by nesidioblastosis. However, this approach is not recommended since PPH is primarily due to alterations in digestive anatomy rather than pancreatic β-cell proliferation. Despite some success in symptom resolution, partial or complete pancreatectomy carries significant risks, including high postoperative morbidity, mortality, and a high likelihood of symptom recurrence.

Partial pancreatic resection, though controversial and typically reserved for severe cases, has been considered in managing PPH linked to hyperinsulinemia caused by nesidioblastosis (53, 119). However, this approach is not recommended since PPH is primarily due to alterations in digestive anatomy rather than pancreatic β-cell proliferation. Despite some success in symptom resolution, partial or complete pancreatectomy carries significant risks, including high postoperative morbidity, mortality, and a high likelihood of symptom recurrence (118, 119).

A summary of various studies on the treatment of PPH is exhibited in Table 4.

**Table 4:** A summary of various studies on the treatment of postprandial hypoglycemia.

1st author/year	Study design	Type of surgery	Number of subjects	Intervention/comparison	Findings
Nielsen et al. 2022 (122)	RCT cross-over	RYGB	10	Dasiglucagon vs. placebo	Two doses of Dasiglucagon Were administrated (80 and 200 µg). Single-dose administration significantly increased plasma glucose and reduced hypoglycemia time.
Sheehan et al. 2022 (123)	Clinical trial open-label	RYGB	14	Pramlintide (before vs. after)	In patients with post-bariatric hypoglycemia (PBH), pramlintide does not affect glycemic or insulin responses, satiety, or dumping scores during mixed-meal tolerance tests (MMTT). Additionally, it does not alter glycemic fluctuations or reduce low sensor glucose levels in outpatient settings.
Ciudin et al. 2021 (51)	Prospective pilot	RYGB	21 (16 RYGB patients with PHH, 5 healthy controls)	Canagliflozin 300 mg daily for 2 weeks	- Significant reduction in plasma glucose levels during OGTT after treatment with Canagliflozin (minute 30: 161.5 ± 36.22 vs. 215.9 ± 58.11 mg/dl; minute 60: 187.46 ± 65.88 vs. 225.9 ± 85.60 mg/dl, *p* < 0.01). - Significant decrease in insulin levels during OGTT (minute 30: 95.6 ± 27.31 vs. 216.35 ± 94.86 mg/dl, *p* = 0.03; minute 60: 120.85 ± 94.86 vs. 342.64 ± 113.32 mIU/L, *p* < 0.001).—85.7% reduction in the rate of hypoglycemia at minute 180 (*p* < 0.00001).
Tan et al. 2020 (124)	Phase 2, multiple-ascending-dose	RYGB, vertical SG	19 women with PBH	Avexitide (exendin 9–39)—Multiple doses (Lyo avexitide & Liq avexitide)	- Treatment with Lyo avexitide reduced the magnitude of symptomatic hyperinsulinemic hypoglycemia at all dose levels, with dose-dependent improvements in glucose nadir, insulin peak, and symptom score. - Liqavexitide 30 mg BID significantly increased glucose nadir (+47%), reduced insulin peak (−67%), and reduced overall symptom score (−47%). - Both formulations were well tolerated.
Øhrstrøm et al. 2020 (125)	RCT Cross-over	RYGB	11	Acarbose, sitagliptin, verapamil, liraglutide, pasireotide	- Treatment effects were evaluated by mixed-meal tolerance test (MMTT) and, for all except pasireotide, by 6 days of continuous glucose monitoring (CGM). - Acarbose and pasireotide significantly increased nadir glucose levels and reduced time in hypoglycemia during MMTTs. - Acarbose decreased peak glucose levels, whereas pasireotide increased both peak glucose and time in hyperglycemia. - Verapamil and liraglutide had no significant impact on hypoglycemia. - Pasireotide significantly diminished glucagon-like peptide-1 (GLP-1) levels.
Øhrstrøm et al. 2019 (126)	Cross-over	RYGB	5	Pasireotide (75 μg, 150 μg, 300 μg)	- Administered as a single dose of varying amounts (75 μg, 150 μg, 300 μg).—All doses prevented hypoglycemia but resulted in notable increases in postprandial hyperglycemia. - Pasireotide significantly diminished insulin, C-peptide, and GLP-1 responses. - The 75 μg dose appears sufficient to prevent hypoglycemia in RYGB-operated individuals with PBH, with reduced hyperglycemia compared to higher doses.
Salehi et al. 2014 (112)	RCT	RYGB	9 GB patients with hypoglycemia, 7 GB patients without hypoglycemia and 8 controls	GLP-1 receptor (GLP1R) antagonist, exendin-(9–39)	GLP-1 receptor significantly corrected post-prandial hypoglycemia in GB patients.
Abrahamsson et al. 2013 (47)	Open treatment, uncontrolled observations	RYGB	5	GLP-1 analogs	- Five consecutive GBP cases with late postprandial hypoglycemic symptoms were treated with GLP-1 analogs. - Symptoms were eliminated in all cases, with relapses occurring when treatment was reduced/discontinued. - Continuous glucose monitoring in one case further documented the drug's effect. The study suggests GLP-1 analogs as a new treatment option for late PPHG.
Plamboeck et al. 2013 (50)	Observational	Truncal vagotomy with pyloroplasty	20 vagotomized subjects, 10 healthy controls	Infusions of GLP-1 or saline, then comparison of food intake, gastric emptying, insulin, and glucagon responses	- GLP-1 reduced food intake in control subjects but not in vagotomized subjects. - GLP-1 slowed gastric emptying in controls but had no effect in vagotomized subjects. - Higher peak postprandial GLP-1 levels in vagotomized subjects. - GLP-1 reduced insulin secretion in controls but did not affect vagotomized subjects. - GLP-1 reduced glucagon secretion in both groups, but levels were about twice as high in vagotomized subjects and nonsuppressible in the early phase.
Ritz et al. 2012 (127)	Pilot	RYGB	8	Dietary counseling + acarbose	- Patients with dumping syndrome are treated with dietary counseling and acarbose (50–100 mg three times a day). - Symptoms disappeared in seven out of eight patients. - Significant increase in the time to interstitial glucose (IG) peak, reduced rate of IG increase and decrease after a meal. - Significant decrease in time below 60 mg/dl (from 2.5% to 0.18%), and increased minimum IG levels.
Speth et al. 1983 (128)	RCT Cross-over	Billroth II and truncal vagotomy with pyloroplasty	9	Acarbose, pectin, a combination of acarbose with pectin vs. placebo	- 4.2 grams of pectin reduced post-meal peak glucose (*p* < 0.01). - Acarbose (50 mg) and its combination with pectin increased plasma glucose 60–150 min post-meal (*p* < 0.01, *p* < 0.05, respectively). - The combination of acarbose and pectin reduced plasma insulin peaks (*p* < 0.05). - Hypoglycemia symptoms were common: eight out of nine patients were on placebo, two on 50 mg acarbose, two on 100 mg acarbose, five on pectin, and two on the combination. - All acarbose treatments increased breath hydrogen (*p* < 0.05).

RYGB, Roux-en-Y gastric bypass; SG, sleeve gastrectomy; RCT, randomized controlled trial; GB, gastric bypass; PHH, postprandial hypoglycemia; MMTT, mixed-meal tolerance test; OGTT, oral glucose tolerance test; PBH, post-bariatric hypoglycemia; GLP-1, glucagon-like peptide-1.

## 9 Conclusion

In summary, PPH following bariatric and metabolic surgery presents with a range of neuroglycopenic and adrenergic symptoms, and its diagnosis remains challenging due to the lack of standardized clinical guidelines. The primary approaches to managing PPH following bariatric and metabolic surgery include Lifestyle Modifications, Exercise, and Nutrition Management. The literature recommends implementing dietary changes, such as limiting carbohydrates, avoiding high glycemic index foods, opting for heart-healthy fats and sufficient protein, refraining from alcohol and liquids during meals, and adjusting meal timing. Pharmacotherapy is essential when lifestyle modifications and dietary changes are insufficient. Surgical interventions are considered a last resort for patients who do not respond sufficiently to dietary, medical, or other non-surgical treatments. Further investigations into predictive markers, optimal treatment strategies, and long-term outcomes will be pivotal in refining our approach to mitigating the impact of this challenging complication on postoperative patients. These efforts will enhance our ability to effectively manage PPH and improve the quality of life for those affected.
